# The circadian clock modulates *Anopheles gambiae* infection with *Plasmodium falciparum*

**DOI:** 10.1371/journal.pone.0278484

**Published:** 2022-12-01

**Authors:** Tibebu Habtewold, Sofia Tapanelli, Ellen K. G. Masters, Nikolai Windbichler, George K. Christophides

**Affiliations:** Department of Life Sciences, Imperial College London, London, United Kingdom; Universidade Federal do Rio de Janeiro, BRAZIL

## Abstract

Key behaviours, physiologies and gene expressions in *Anopheles* mosquitoes impact the transmission of *Plasmodium*. Such mosquito factors are rhythmic to closely follow diel rhythms. Here, we set to explore the impact of the mosquito circadian rhythm on the tripartite interaction between the vector, the parasite and the midgut microbiota, and investigate how this may affect the parasite infection outcomes. We assess *Plasmodium falciparum* infection prevalence and intensity, as a proxy for gametocyte infectivity, in *Anopheles gambiae* mosquitoes that received a gametocyte-containing bloodfeed and measure the abundance of the midgut microbiota at different times of the mosquito rearing light-dark cycle. Gametocyte infectivity is also compared in mosquitoes reared and maintained under a reversed light-dark regime. The effect of the circadian clock on the infection outcome is also investigated through silencing of the *CLOCK* gene that is central in the regulation of animal circadian rhythms. The results reveal that the *A*. *gambiae* circadian cycle plays a key role in the intensity of infection of *P*. *falciparum* gametocytes. We show that parasite gametocytes are more infectious during the night-time, where standard membrane feeding assays (SMFAs) at different time points in the mosquito natural circadian rhythm demonstrate that gametocytes are more infectious when ingested at midnight than midday. When mosquitoes were cultured under a reversed light/dark regime, disrupting their natural physiological homeostasis, and infected with *P*. *falciparum* at evening hours, the infection intensity and prevalence were significantly decreased. Similar results were obtained in mosquitoes reared under the standard light/dark regime upon silencing of *CLOCK*, a key regulator of the circadian rhythm, highlighting the importance of the circadian rhythm for the mosquito vectorial capacity. At that time, the mosquito midgut microbiota load is significantly reduced, while the expression of *lysozyme C-1* (*LYSC*-1) is elevated, which is involved in both the immune response and microbiota digestion. We conclude that the tripartite interactions between the mosquito vector, the malaria parasite and the mosquito gut microbiota are finely tuned to support and maintain malaria transmission. Our data add to the knowledge framework required for designing appropriate and biologically relevant SMFA protocols.

## Introduction

The yearly downturn trend in malaria cases and deaths has been plateauing since 2015, especially in the most burdened countries of sub-Sahara Africa [1]. This stagnation is caused not only by insufficient coverage with insecticide treated nets (ITNs) and indoor residual spraying (IRS), but also by the selection of mosquito populations that mediate outdoor malaria transmission and/or exhibit multiple insecticide resistance traits. Presently, additional approaches to complement ITNs and IRS are being tested, including some that aim to block the transmission of the *Plasmodium* parasite to and from the mosquito vectors [2, 3]. Such transmission blocking technologies must pass through rigorous testing before they can be implemented [4].

The development of the *Plasmodium* parasite inside an *Anopheles* mosquito begins with the uptake of gametocytes, the sexual form of the parasite, upon a bloodmeal. Gametocytes are rapidly activated in the mosquito gut lumen to produce gametes that fuse to form a zygote that further develops into a motile ookinete. Within 24h after the bloodmeal, the ookinete invades the mosquito midgut epithelium and, upon reaching the basal lamina, forms an oocyst. Inside the oocyst, the parasite undergoes multiple rounds of endomitotic replications followed by the formation of thousands of sporozoites, a process known as sporogony. When the oocyst ruptures, the sporozoites are released into the hemolymph and invade the salivary glands for transmission upon a subsequent mosquito bite [5]. To date, transmission blocking technologies are mostly aimed at inhibiting gametocyte mosquito infectivity but targeting parasite sporogonic development has also gained traction in recent years. Thus, fully understanding and emulating the natural settings by which a mosquito infection can lead to transmission is critical for the development and evaluation of such technologies.

Enumeration of oocysts in the midgut of mosquitoes, typically 7–10 days after laboratory mosquito infection, is considered as one of the gold standard assays for prioritizing the most promising transmission blocking technologies. Current methods for achieving the laboratory mosquito infection includes offering the mosquitoes a bloodmeal on *in vitro* cultured *P*. *falciparum* gametocytes or blood directly drawn from a gametocytemic patient via an artificial membrane system, and rarely by allowing laboratory reared mosquitoes to feed directly on a patient [6–8]. These methods largely overlook some critical environmental variables that may have a significant impact on the outcome of the mosquito-parasite *interaction and malaria transmission* [9, 10]. Indeed, a key natural environmental determinant of this interaction is the light-dark rhythm [11–14].

In their natural environment, mosquitoes are directly exposed to daily fluctuations in light, temperature, and resource availability. Such external stimuli, specifically the 24-h light/dark cycle entrains the mosquito circadian rhythm, including physiological, immunological, and behavioural activities according to their temporal niches. Studies in *Anopheles gambiae* mosquitoes have established the presence of circadian rhythmicity of important behaviours including foraging, mating, and oviposition [15–17]. Accumulating evidence also points to environmental cues affecting the outcome of host-pathogen interaction by regulating the expression of genes involved in such host activities [18–21]. For example, increased expression of key mosquito genes involved in the detoxification of by-products from bloodmeal processing during the night hours when the mosquito naturally ingests a bloodmeal, such as *Catalase* that reduces a number of reactive oxygen species (ROS), enhances the proliferation of the mosquito gut microbiota that has major impacts on the parasite infection [22].

The expression of genes involved in the immune response is rhythmically oscillatory throughout the day. The link between rhythms in the abiotic environment, specifically photoperiods and the insect innate immune response was first investigated in *Drosophila melanogaster*. Flies kept in a 12:12 light-dark (LD) condition and challenged with *Streptococcus pneumoniae* at 7 hours after the start of the light phase (zeitgeber time 7, ZT7), exhibited a lower survival rate compared to flies challenged at ZT19, 7 hours after the start of the dark phase [23]. Further studies have conducted various experiments to determine the correlation between circadian rhythms and innate immunity [24–26].

In *A*. *gambiae*, the expression of nearly 50 immune genes, including those involved in the immune deficiency (Imd) and melanisation pathways, was reported as being affected by the circadian rhythm [27]. Similarly, in *A*. *stephensi*, antimicrobial peptides (AMPs) *Defensin-1* (*DEF-1*) and *Cecropin-1* (*CEC-1*) as well as *nitric oxide synthase* (*NOS*) show circadian rhythmicity [28]. The mosquito is thought to rhythmically regulate its immune responses as constant activation of immunity can result in unwanted fitness costs whereas a circadian-fashioned immune response would allow immune pathways to increase their activity only when it is most likely to be required [28, 29]. This rhythmic immune response means that the mosquitoes may be more susceptible to infection at a certain time of the day. Asexual development of the *Plasmodium* parasite also exhibits a daily rhythm that affects the expression of a significantly large number of genes and cell cycle progression including gametocyte development [30, 31]. Importantly, *P*. *falciparum* has shown a persistent rhythmicity even in the absence of the host rhythm, *i*.*e*. when cultured *in vitro*, although it can quickly lose populational synchrony, suggesting that both host cues and a parasite internal timekeeping mechanism are involved in driving this rhythm [32–34]. Therefore, one may hypothesize that how the two rhythms align would very much determine the infection outcome. On one hand, the parasite rhythm allows it to take advantage of the mosquito rhythmicity to enhance its infectivity, while on the other hand, the mosquito rhythm enables anticipation of parasite infection and readiness of its immune defence.

The above phenomena underline the importance of emulating the natural setting when performing laboratory infections when studying vector-parasite interactions to derive the most meaningful conclusions. This observation contrasts with most laboratory experimental conditions, where infections with cultured *Plasmodium* gametocytes occur during daytime, leading to a mismatch between the mosquito rhythms driven by external stimuli (e.g. photoperiod) and the parasites. As part of our effort to develop a synchronised-dark-feeding system for consistent high intensity infections of *A*. *gambiae* with *P*. *falciparum* NF54 laboratory model, we observed that gametocyte infectivity and mosquito infection prevalence were significantly higher when mosquitoes were kept in the dark 3h before blood feeding and maintained in these conditions for 25h post-infection compared to mosquitoes maintained and fed during the day hours maintained in a normal light cycle [35]. Here, we extended this study to investigate the effect of light/dark phases on the infection outcome.

## Materials and methods

### Mosquito rearing

*Anopheles coluzzii* mosquitoes (previously known as *A*. *gambiae* M form) of the N’gousso strain were reared and maintained under normal insectary conditions, *i*.*e*. 27 ± 1°C and 70 ± 5% humidity on a light-dark (LD) cycle: 11.5h full light of approximately 300 lux starting at 6am and 11.5h darkness starting at 6pm, with 30 min dawn and dusk transitions, respectively. For photoperiod manipulation, mosquitoes were reared throughout their development in the insectary in plastic boxes (1m L x 0.75m W x 0.5m H) wrapped in aluminium foil to prevent light exposure. A LED 6W lamp (about 300 lux) regulated by an independent light switch timer was installed in the box. In one of the boxes, the time of light-on (L) and -off (D) was set to the same zeitgeber time (ZT) as the normal insectary regime (where the light and dark phases start after 6am and 6pm, respectively; LD 12:12), and, in another second box, the controller was timed to switch-on and -off opposite to the insectary regime (i.e. the light and dark phase start after 6pm and 6am, respectively; DL 12:12) exposing mosquitoes to a reverse zeitgeber time of the original rearing regime. To disrupt the circadian rhythm, the mosquitoes were reared and maintanied under a constant light (LL) (to abolish the rhythm all together [36]) or constant darkness (DD).

#### Gametocyte culturing

The *P*. *falciparum* NF54 strain (MRA-1000, patient E) was used in all experiments reported here. Gametocyte preparation for mosquito infection was performed as previously described [35]. On the day of infection, the gametocyte culture (14 to 16 days old) was assessed for maturation and overall vigour by observing stained smears and exflagellation. The cultures were combined and spun at 500g for 6 min, the resulting pellet was mixed with pre-warmed (37°C) fresh cRBCs (50% vol of the pellet) and pre-warmed (37°C) fresh heat-inactivated human serum (50% vol. of the total volume). The mixture was loaded in pre-warmed (37°C) membrane feeders for standard membrane feeding assay (SMFA).

### Mosquito infection

Four- to five-days old female mosquitoes were potted in 16 oz paper cups (n = 60), sealed with a double layer net, and starved for 3-4h before infection. The mosquitoes were allowed to bloodfeed on the membrane feeder. Routinely, the mosquito infection with *P*. *falciparum* takes place in designated room at a light intensity of 0 lux (has a dim light which enters the room via a 30 cm^2^ tinted glass window) to improve the mosquito blood intake. The infection is completed within 10–15 min, hence bias due to a mismatched light/dark phase remans insignificant. Following the infection, the mosquitoes were kept in the incubator set at 27°C with 60–70% humidity at their respective light-dark cycle until 48h post infection when the transformation of all viable ookinete to oocyst is completed [37]. ZT of mosquito infection varied with specific experiments and are provided in the relevant sections. Sugar solution was provided to the mosquitoes 48h after infection to starve and kill unfed individuals.

On day 7 post-infection, the mosquitoes were killed with ethanol and midgut dissection was performed in ice cold PBS. The midguts were stained with mercurochrome (0.5%) for 20 min and fixed in 4% paraformaldehyde for 30 min. Oocysts were visualized and counted using a light microscope at 20X magnification.

#### Measuring the size of blood bolus

To investigate whether the ZT of mosquito infection affects the size of bloodmeal uptake, 10 freshly fed mosquitoes were dissected and their midguts were individually homogenised in 1 mL PBS. A volume of 10 mL of the homogenate was loaded into a Fast-Read 102® plastic counting chamber, and RBCs in four squares were counted to calculate the total number of cells per midgut.

#### Gene silencing

The mosquito *CLOCK* (AGAP005711) gene was silenced by RNAi following double-stranded RNA (dsRNA) injection in the mosquito hemocoel. To synthesize the dsRNA, a fragment of the target gene was PCR amplified using gene-specific primers (F: CTAGCGGTGGTTCGTTTCTG and R: GTAAAGTTGAGCTGCTCCGG) flanked by the short T7 promoter sequence TAATACGACTCACTATAGGG from a cDNA library made from mRNA extracted from mosquito tissues. The dsRNA production was performed using the MEGAscript T7 Kit (Ambion, UK). Purified dsRNA (using RNeasy kit, QIAgen®, UK) was concentrated to 3 μg/μl. Three days before infection, dsRNA (69nl) was injected into the lateral side of the thorax of 0-1-day old female mosquitoes; control mosquitoes were injected with dsRNA of the *LacZ* gene (*dsLacZ*). Ten mosquitoes were subsampled before infection to extract tRNA which was used as template to synthesize cDNA. The cDNA was used in the RT-qPCR reaction to assess the level of gene silencing. Mosquito infections were carried out between ZT7-8.

#### Quantification of the midgut microbiota

Mosquito midgut microbiota were measured as previously described [38]. Briefly, 10 midguts were dissected out from 3-5-day old female mosquitoes maintained in the LD or DL regime on sugar only. Midgut tissues were dissociated in 800 μl Gibco® Cell Dissociation Buffer (ThermoFisher Scientfic, UK) for 10 min on a 37°C shaker. The samples were briefly vortexed and stained with LIVE/DEAD BacLight assay kit according to the manufacturer (ThermoFisher Scientific, UK). The mixture was incubated at room temperature for 10 min and centrifuged for 10 min at 4°C and the resulting pellet was resuspended in 1 mL ice-cold PBS. A volume of 25 μL of bead suspension (containing 990 beads/μL) was added to the sample before flow cytometry (FCM) analysis to determine the number of bacteria per microliter of gut homogenate. The total bacterial count per gut was calculated as the ratio of the number of events in the bacterial cell population and the number of events in the bead population, multiplied by the ratio of the total number of beads used in the final volume of the test sample.

### Measuring *Lysozyme* (*LYSC-1)* gene expression

To assess the expression of Ag*LYSC-1* (AGAP007347), midguts were dissected from 3–4 days old female mosquitoes at ZT0, ZT6, ZT12 and ZT18 (n = 15). Total RNA extracted from individual midguts and used to synthesis cDNA to be used as template in the RT-PCR reaction to amplify Ag*LYSC-1* using gene-specific primer sets (F: TCAAGTGTGCCAAGCTGATCCAC and R: GCGTCCTTAAAAACAGGAGCTA). Experiments were repeated twice. The total volume of reagents was 20 μl containing 2 μl cDNA, 10 μl of 2× Fast SYBR™ Green PCR Master Mix protocol (Thermo Fisher Scientific, UK) and 200 nM of each primer. Amplification and detection of fluorescence signals were carried out using an Applied Biosystems 7500 Fast Real-Time PCR system. The PCR cycling program consisted of an initial denaturation stage at 95°C for 20 s, followed by 40 cycles at 95°C for 3 s and 60°C for 30 s. Each gene was quantified in duplicate, and the threshold crossing values (C_T_-values) were standardised using a standard curve and normalised to the geometric mean of the S7 rRNA gene that served as an internal control.

### Statistical analysis

All statistical analyses were performed using the GraphPad Prism 9 software. The comparison of oocyst intensity data was performed using the Kolmogorov-Smirnov test, whereas the infection prevalence was compared using the *χ*^2^ test with Yates correction. Microbiota count data were analysed with the Kruskal-Wallis test using the Conover-Inman method for pairwise contrasts between time points.

## Results

### Gametocyte infectivity is higher at night-time

**Fig 1:**
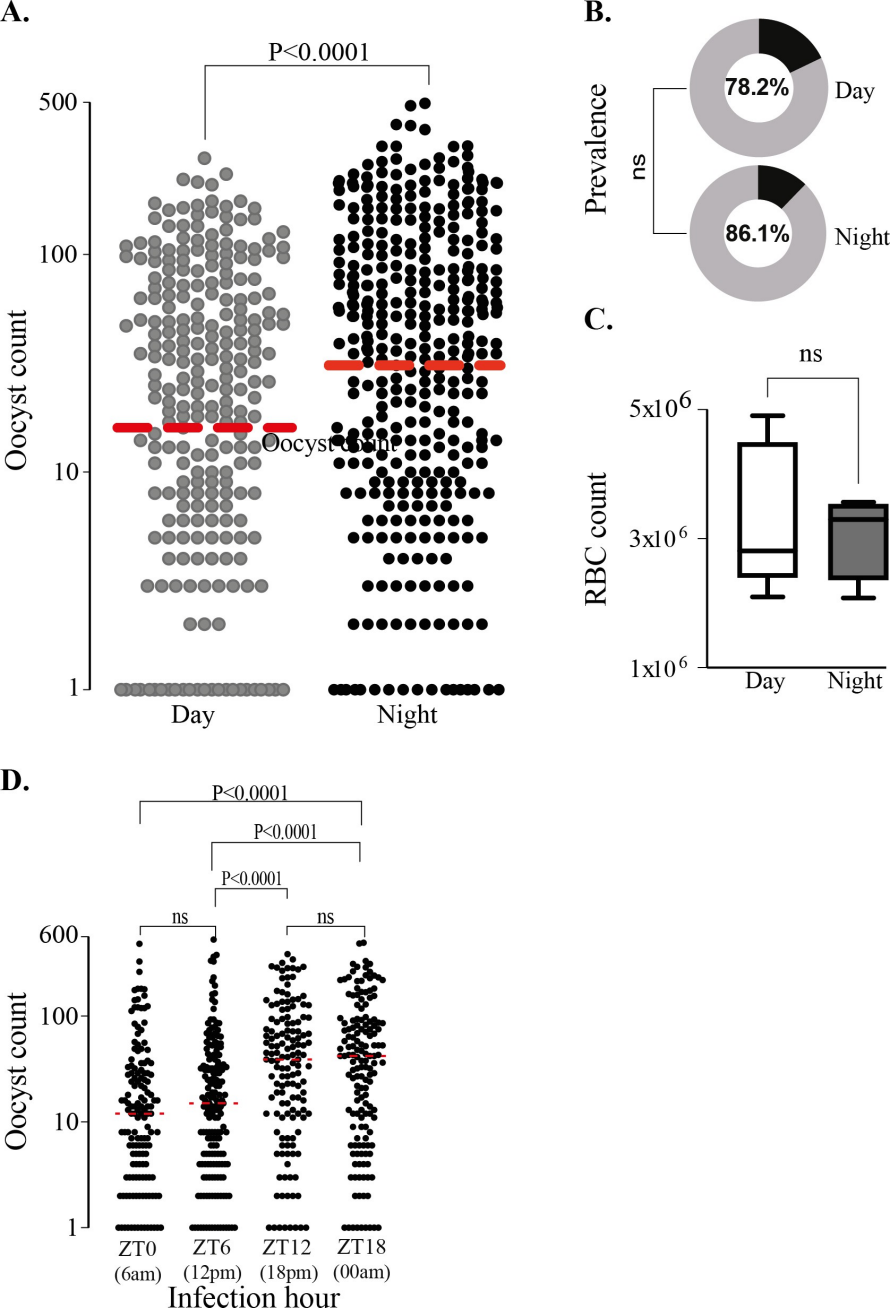
*Plasmodium* infectivity at day-time vs night time. (**A**) Oocyst intensity in mosquitoes received bloodmeal containing gametocyte from same culture flask at day-time (7 ZT) vs night-time (14 ZT); (**B**) Oocyst infection prevalence in day-time and night-time. The data in A&C were from five replicates while B were from two replicates. Midguts dissection for oocyst examination was carried out on day-7 post infection (pi). (**C**) number of RBCs (millions) in the blood bolus isolated from freshly fed mosquitoes; Box represents the 25th to 75th percentiles and whiskers represent the mininum and maximum values. (**D**) Oocyst infection intensity in mosquitoes received gametocyte at different ZT.

*P*. *falciparum* oocyst intensities in the midgut were compared between mosquitoes infected at daytime (ZT7; *i*.*e*. 13:00pm) vs evening time (ZT14; *i*.*e*. 20:00pm) using gametocytes originating from the same culture-flask (**Fig 1A**). The results showed a significantly higher oocyst load at ZT14 compared ZT7 (P<0.0001). Oocyst prevalence, the proportion of midguts harbouring one or more oocysts, was not statistically different between the two cohorts (**Fig 1B**). Measurements of bloodmeal uptake showed no significant difference between the two mosquito groups (**Fig 1C**), suggesting that the observed differences in oocyst load was due to the time of infection and not the bloodmeal volume, although the variation was higher during daytime.

The results prompted us to further investigate the circadian variation in infection outcome in infections performed at ZT0 (6:00am), ZT6 (12:00pm), ZT12 (18:00pm) and ZT18 (00:00am). Four flasks of gametocyte cultures were pooled and split between four new flasks 24h before the first feed, and one flask was used for each feeding timepoint. In four independent experiments, the infection intensity varied between mosquito groups (**Fig 1D**). Statistical analysis showed that the oocyst count per midgut was significantly higher in mosquitoes infected at ZT18 (00:00am) compared to all other timepoints (P<0.0001 in each pairwise comparison). We caution readers about lower sample size included in the mosquitoes infected at ZT18 due to an increased mortality in this group [39]. No other pairwise comparisons were statistically significant. Observation of a similar oocyst intensity at ZT12 and ZT18 suggests that the dark phase functions are initiated in the mosquitoes as early as ZT12 and the mosquito susceptibility to *P*. *falciparum* (parasite’s normal development process) is optimal in the evening. Our finding suggests that recent shift in biting habits of malaria vectors, early-night and late-night biting (*e*.*g*. [40]) would not impact mosquito susceptibility to the parasite.

### Circadian rhythm regulates the midgut microbiota and AMP expression

**Fig 2:**
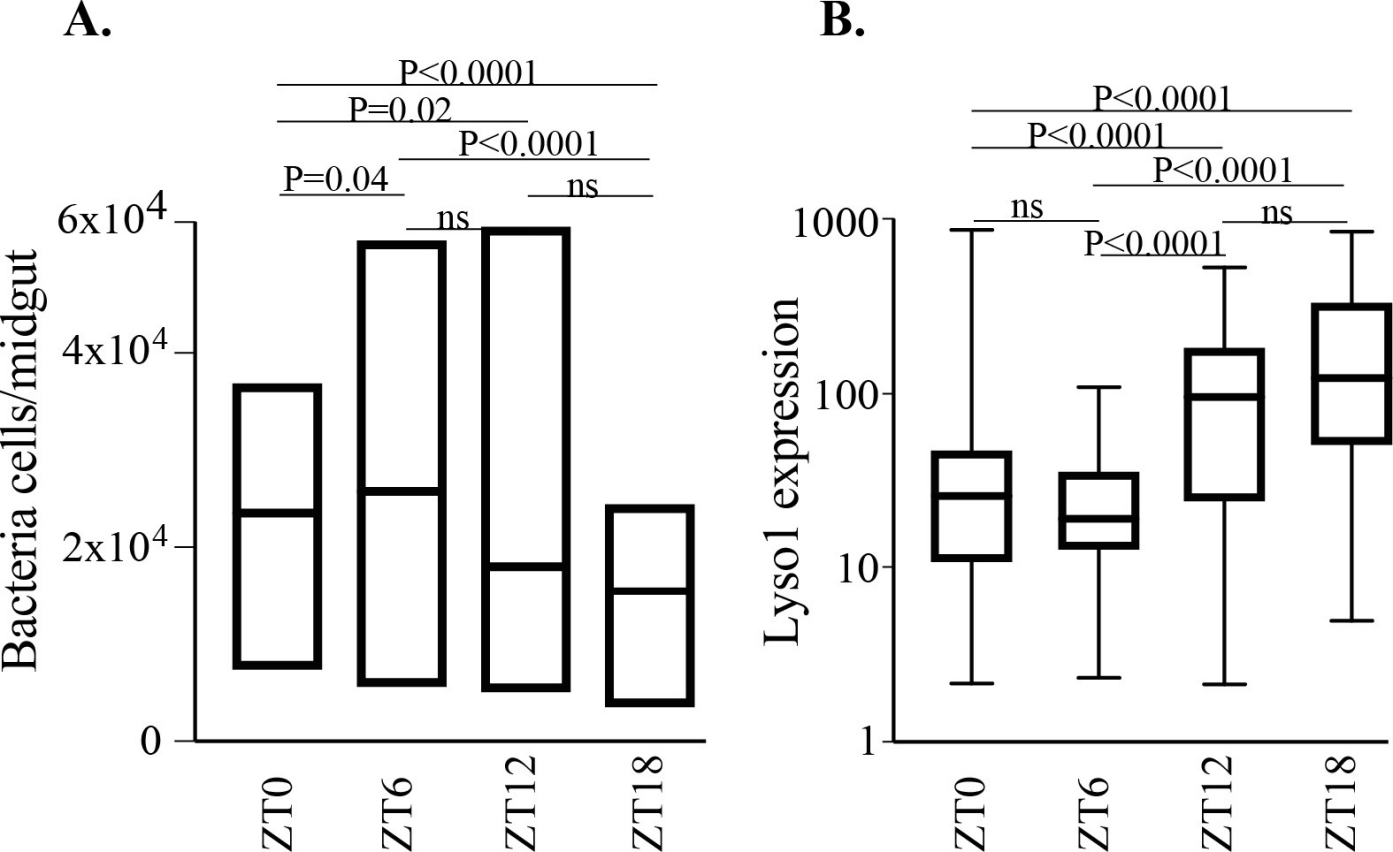
Diurnal dynamics of mosquito midgut microbiota and the expression of antibacterial gene *lysozyme c-1* (*LYSC-1*). Floating bars (A) represent maximum to minimum microbiota count at different timepoints, where the horizontal bar represents the median count, and box plot (B) represent the level of relative expression (ng/mosquito) of *LYSC-1* determined using RT-qPCR on tRNA extracted from mosquitoes sampled at different ZT.

A robust body of literature[41–44] describes a three-way interaction between the vector, the parasite and the microbiota, which impacts gametocyte infectivity of the mosquito and, ultimately, the infection outcome. This prompted us to investigate the diurnal rhythmicity of the mosquito midgut microbiota. We measured the load of midgut microbiota using flow cytometry (FCM) at the four different timepoints, *i*.*e*. ZT0 (06:00am), ZT6 (12:00pm), ZT12 (18:00pm) and ZT18 (00:00am), in 5-day-old female mosquitoes maintained on sugar under a regular insectary lighting regime. The results showed a significantly lower microbiota load at ZT18 compared to ZT0 (P<0.0001) and ZT6 (P<0.0001) (**Fig 2A**). The microbiota load at ZT12 and ZT6 were similar. In addition, the microbiota load at ZT0 was significantly lower than ZT6 (P<0.04), but significantly higher than ZT12 (P<0.02). In summary, microbiota abundance at ZT18<ZT0<ZT6 or <ZT12, whereas ZT6 = ZT12. Importantly, the reduced microbiota load at ZT18 coincided with a significantly elevated expression of *LYSC-1*in the evening hours, i.e. ZT12 and ZT18 (**Fig 2B**), an antimicrobial peptide that hydrolyses bacterial cell wall leading to cell lysis [45]. *LYSC-1* expression at ZT12 and ZT18 were higher compared to ZT0 (P<0.0001) or ZT6 (P<0.0001), whereas the expression level at ZT12 and ZT18 or ZT0 and ZT6 were similar.

### The daily rhythm of mosquito on the *Plasmodium* infectivity

**Fig 3:**
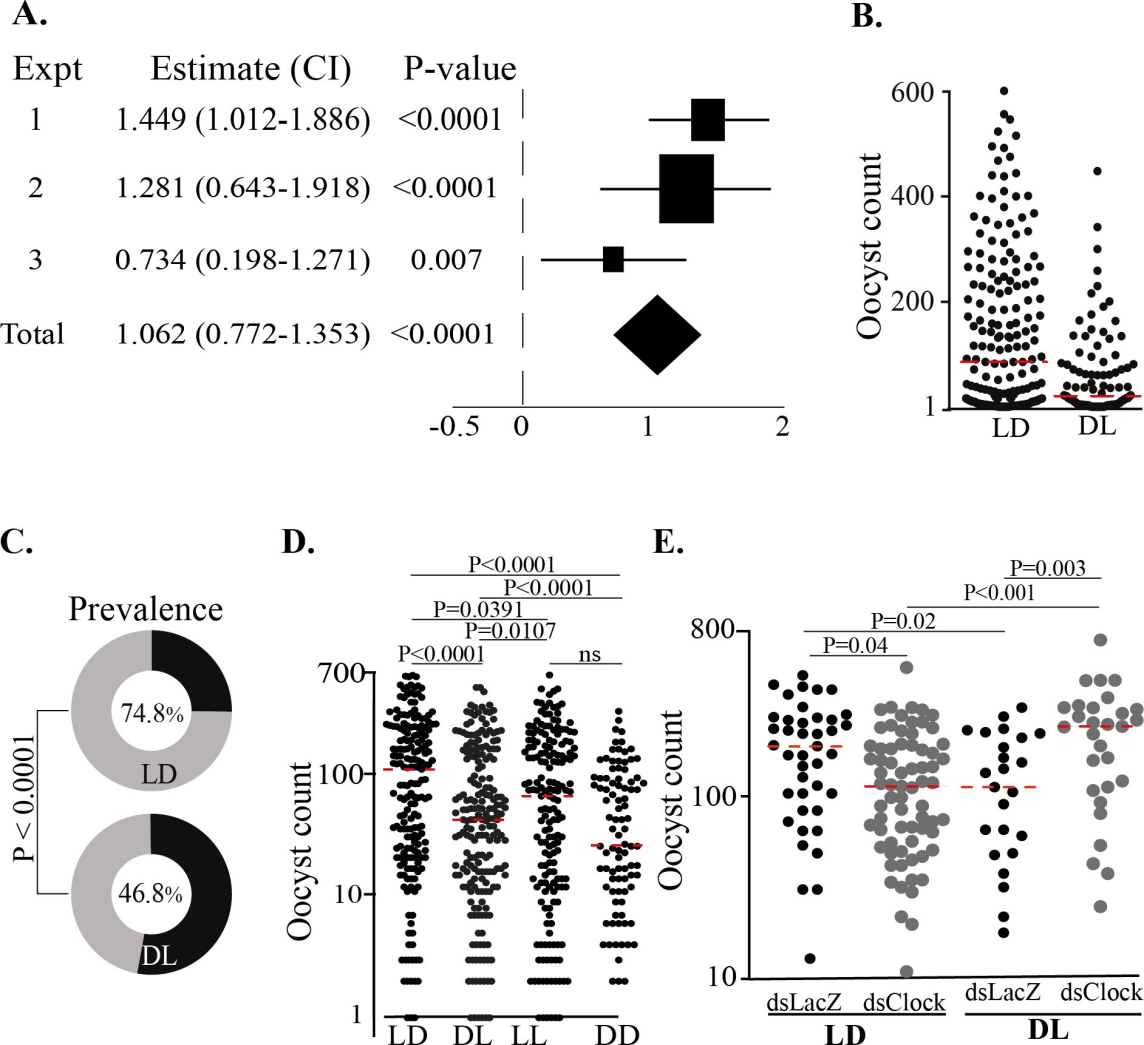
Mosquito circadian rhythm on the *P*. *falciparum* gametocyte infectivity. (**A**) Forest plot showing an estimate of effect ratio (± 95% CI) of oocyst intensities between mosquitoes under a LD regime and DL regimes; Squares and diamond show the sample size for each replicate and the total, respectively. (**B**) Overall oocyst intensities between mosquitoes under a LD and DL regimes; Red line represents the median. (**C**) Pie-charts showing oocyst infection prevalence in mosquitoes under LD and DL light-dark regime. The plots represent data from three independent replicates. (**D**) Oocyt intensity in the midguts of mosquitoes reared and maintanied under differnet light/dark regime: (LD) the standard insectary ligh (12h)/dark (12h), (DL) reversed dark/light regime, (LL) constant light or (DD) constant dark. (**E**) Effect of CLOCK gene silencing on the oocyst intensity in mosquitoes under normal and reversed light/dark cycle. Red lines represent the median.

To exclude the possibility that other factors in the insectary during daytime or the time difference in the use of the gametocyte culture influence the observed *P*. *falciparum* infection outcomes, we created two experimental mosquito groups, each reared, maintained in opposite light-dark regimes: LD (L starting at 6am and D starting at 6pm) and DL (L starting at 6pm and D starting at 6am). Both groups were infected at 7pm, that being ZT13 for LD and ZT1 for DL mosquitoes, which allowed us to use gametocytes from the same culture flasks and to sample at the same time. The results confirmed that mosquitoes in the LD regime exhibited a significantly higher infection than those in the DL regime, in terms of both oocyst load (**Fig 3A** and **3B**; P<0.0001) and prevalence (**Fig 3C**; P<0.0001), confirming the strong impact of the mosquito circadian rhythm on *P*. *falciparum* infection outcome. The results of comparison of oocyst intensity in mosquitoes reared and maintanied under differnet photonic regime: LD, DL, LL, or DD showed that the mosquito under LD regime had higher oocyst load (**Fig 3D**) compared to DL (P<0.0001), LL (P = 0.0391) or DD (P<0.0001), and the oocyst intensity was in LL mosquito was significantly higher compared to LD (P = 0.0107) or DL (P<0.0001), but the infection intensity in DL and DD mosquitoes were not significantly different.

Next, we investigated whether this pattern of infection changes when the mosquito circadian rhythm is deregulated. We used dsRNA injections in the mosquito hemocoel to silence the *A*. *gambiae CLOCK* gene (AGAP005711), one of the core circadian clock genes, in mosquitoes reared under the LD or the DL regime and infected with *P*. *falciparum* gametocytes at 7pm as above: ZT13 for LD and ZT1 for DL mosquitoes. Mosquitoes of both groups were injected with dsRNA of the *LacZ* gene as a control. RT-PCR performed on total RNA extracted from *dsCLOCK* mosquitoes showed 37.9% silencing (data not shown). The results were striking, showing that *CLOCK* silencing can reverse the parasite infection outcome (**Fig 3E**). Specifically, LD mosquitoes showed a significantly reduced oocyst load when *CLOCK* was silenced (P = 0.005), whereas in DL mosquitoes *CLOCK* silencing led to a higher oocyst load (P = 0.003), each compared to their respective *dsLacZ*-injected controls. Furthermore, *CLOCK*-silenced DL mosquitoes exhibited significantly higher oocyst loads compared to *CLOCK*-silenced LD mosquitoes (P = 0.0005). In control dsLacZ-injected mosquitoes, the oocyst load was significantly higher in LD than DL mosquitoes (P = 0.02).

## Discussion

The research reported here is an extension and in-depth investigation of observations made in a previous study where we showed that *A*. *gambiae* mosquitoes kept in dark conditions for at least 3 hours prior to ingestion of *P*. *falciparum* gametocytes exhibit higher infection intensity and prevalence than mosquitoes kept in light conditions [35]. We assessed the impact of the mosquito circadian clock on the infection outcome and demonstrated that the infection intensity peaks during the night, a time that corresponds to the natural feeding period of *A*. *gambiae* mosquitoes. Previous studies have reported an increased infectivity of *P*. *falciparum* gametocytes during the natural foraging hours of the mosquito [29, 46, 47] and in the *Plasmodium relictum* and *Culex pipiens* system, increased mosquito infection was reported in the late afternoon, which coincides with natural biting hours of the vector [48], suggesting that vector daily rhythms have a role in shaping the interactions between the vector and *Plasmodium* parasite. Studies have shown that a considerable proportion (>20%) of *A*. *gambiae* genes have rhythmic expression, including immunity-related genes such as those encoding regulators of the IMD pathway and immune effectors including *defensin 1* (*DEF1*), *cecropin 1* (*CEC1*) and *nitric oxide synthase* (*NOS*) [28], which may therefore contribute to the differential infection susceptibility of mosquitoes across the diel cycle [18, 27].

We have shown that the abundance of midgut microbiota in *A*. *gambiae* was altered in a time-of-day-specific manner with the lowest abundance recorded at midnight, compared to midday. This is attributable to variations in the mosquito antimicrobial response across 24h, which is achieved through two possible pathways. The first is lysozyme-mediated bacterial killing and digestion as food. This is supported by observations in this and previous studies, which demonstrate an elevated level of c-type lysozyme expression during the night [27]. Canonically, the lysozymes kill bacterial cells by hydrolysing the cell wall peptidoglycan and thereby playing part both in digestion and immune defence [49]. The second pathway involves reactive oxygen species (ROS)-induced microbial killing. For example, evidence indicates that *A*. *gambiae* mounts an elevated level of ROS during *the* night phase when the mosquito forages [27, 50, 51]. In both *A*. *gambiae* and *A*. *stephensi* mosquitoes, it was shown that heme-peroxidases catalyse H_2_O_2_-dependent oxidation and the product of this process directly retards microbial metabolism and growth [52, 53]. Midgut microbiota is a cornerstone of innate immunity. For instance, elimination of gut bacteria by treating mosquitoes with an antibiotic cocktails leads to a higher *Plasmodium* infection intensity [54]. Thus, rhythmicity of midgut microbiota may have directly or indirectly contributed to the observed temporal variation in the mosquito susceptibility to *P*. *falciparum* gametocyte infection and the roles of midgut microbiota on mosquito susceptibility to *Plasmodium* parasite has been demonstrated in previous studies [55]. Increased ROS in the night induces the production of heme-peroxidases HPX15 (a potent neutraliser of ROS) in the mosquito, which interacts with midgut mucins to prevent immune priming by blocking the interaction between microbiota and epithelial receptors. This protects the mosquito from hyper-immune toxicity and the *Plasmodium* parasite benefits from reduced immune challenges, *e*.*g*. a decreased intensity of *P*. *berghei* and *P*. *falciparum* oocysts was observed in *HPX15*-silenced *A*. *gambiae* [52]. These and other physiological and immunological rhythms in the mosquito vector drive time-of-day specific infectivity of malaria and mismatched infections cause the parasite to suffer fitness costs [32].

Building on this study, we assessed the perturbation of the mosquito rhythm on the *P*. *falciparum* development. Our findings demonstrate that reversing the circadian clock in relation to the time-of-day of gametocyte ingestion in the mosquitoes under dark-light cycle (DL) or abolishing the circadian rhythm all together in the mosquitoes under constant light regime (LL) [36] reduces the ability of the parasite to infect the mosquito. The parasite infectivity was reduced in mosquitoes under constant dark (DD) even though previous studies reported that the circadian rhythms tend to persist under the DD condition in *An*. *gambiae* mosquitoes [27]. Taken all together, we hypothesise that *P*. *falciparum* parasite takes advantage of the natural rhythm of the vector to maximise its vectorial capacity. It has been widely accepted that the parasites’ intrinsic rhythmicity is driven and maintained by the host’s rhythms, which served as cues for the parasites [20, 56]. We hypothesise that a phase shift in circadian rhythms in the DL mosquitoes give cues to the parasite that are out of normal phase expected in natural vector-parasite interactions and hampers the parasite’s ability to coordinate its development patterns to evade danger and exploit resources for a successful transmission. This is also demonstrated by our results where the parasite has a significantly reduced success to infect mosquitoes that were under the LL or DD regime. A previous study in zebrafish larvae demonstrated that response to infection by the larvae was light-regulated [57]. Our finding supports the hypothesis that fine-tuned mosquito–parasite coevolutionary dynamics involve the vector’s investment on its immune response and the parasite’s ability to suppress (or evade) the immune response and exploit resources inside the mosquito [58, 59].

Circadian clock genes are implicated in the variation of daily activity patterns, specifically feeding rhythms in mosquitoes. In the present study, we also demonstrated reduced parasite infectivity in *CLOCK* gene-silenced mosquitoes maintained under the standard light-dark (LD) condition, suggesting that normal mosquito rhythm is essential for increased parasite’s success in the vector. On the other hand, our observation of increased oocyst intensity in mosquitoes under a DL condition and with *CLOCK* silenced is not explainable by existing knowledge, hence warrants further investigations along the line of the microbiota composition, the level of different ROS at systemic and midgut level and also mosquito immune responses. Accumulating literature has shown that the organism’s immune activities including lysozymes expression are under the control of the circadian rhythm of the organism [60, 61]. Although not covered in this study, an investigation into the activity of *LYSC-1* in the *dsCLOCK* silenced or DD mosquitoes should be included in future studies.

Following the introduction of long-lasting insecticide-treated nets (LLINs), a significant shift of biting activity towards the early evening hours has been observed, coinciding with times in which people are outside bed nets [62]. Field-collected *A*. *gambiae* s.l. from Tanzania displayed significant polymorphisms in *clock* genes, without any considerable link with the time and location of their biting activity [63]. It would be interesting to determine the infectivity of wild parasite isolate to these mosquitoes at early hours of the night compared to the preintervention-biting hours. Studies have revealed that endoparasites including *Plasmodium* exhibit a daily rhythm inside vertebrate host, *e*.*g*. a synchronous asexual cycle in *Plasmodium* parasite [64], increased production of microfilaria by the female worm in the evening [19] and the rhythmic expression of metabolic genes in *Trypanosoma* [65] to a maximum exploitation of host’s resources or minimise the impact by host immune responses. These underline the importance of testing mosquito infectivity with wild *P*. *falciparum* collected from gametocyte carriers to emulate the natural vector-parasite interactions in order to derive the most meaningful conclusions.

Currently, there is a strong focus of research on innovative technologies to control mosquitoes and the spread of malaria, for example genetically engineered mosquito strains that will block or limit the transmission of *Plasmodium* parasites by mosquitoes. Critical initial evaluations of such technologies for their efficacy in reducing mosquito infection rely on the laboratory vector–parasite model systems. Most of these do not represent the natural *Anopheles–Plasmodium* interaction, where complex extrinsic and intrinsic factors are involved in the tripartite interactions taking place between the *Anopheles* vector, *Plasmodium* parasite and midgut microbiota, all of which impact the parasite’s survival and establishment [18, 28].

## Conclusion

We conclude that the tripartite interactions between the mosquito vector, the malaria parasite, and the mosquito gut microbiota have been finely and temporally tuned throughout evolution to support and maintain malaria parasitism. In the laboratory systems, the mosquito infection with *Plasmodium* commonly occurs during day-hours, while natural *Plasmodium* transmission occurs at night when vector mosquitoes naturally forage. Our findings underscore the need for adjusting laboratory mosquito infection protocols to emulate, as much as possible, the natural setting. This is critical since testing during this phase informs researchers on the efficacy and safety of novel technology in laboratory populations. This work adds significantly to the knowledge framework required for designing the most appropriate and biologically relevant SMFA protocol to assess whether the new transmission blocking technology demonstrates the molecular, biological, and functional characteristics desired for the chosen application.

## Supporting information

S1 Data (XLSX)
